# Expression of a rice soluble starch synthase gene in transgenic wheat improves the grain yield under heat stress conditions

**DOI:** 10.1007/s11627-018-9893-2

**Published:** 2018-03-06

**Authors:** Bin Tian, Shyamal K. Talukder, Jianming Fu, Allan K. Fritz, Harold N. Trick

**Affiliations:** 1 Department of Plant Pathology, 4024 Throckmorton Plant Sciences Center, Kansas State University, 1712 Claflin Road, Manhattan, KS 66506 USA; 2 Department of Agronomy, 2004 Throckmorton Plant Sciences Center, Kansas State University, 1712 Claflin Road, Manhattan, KS 66506 USA; 3 Samuel Roberts Noble Research Institute, LLC, Ardmore, OK 73401 USA; 4 USDA/ARS/Hard Winter Wheat Genetics Research Unit, 4008 Throckmorton Plant Sciences Center, Kansas State University, 1712 Claflin Road, Manhattan, KS 66506 USA

**Keywords:** Starch synthesis, Wheat transformation, Heat stress, Thermotolerance, Yield

## Abstract

Wheat (*Triticum aestivum* L.) is a temperate cereal with an optimum temperature range of 15–22°C during the grain filling stage. Heat stress is one of the major environmental constraints for wheat production worldwide. Temperatures above 25°C during the grain filling stage significantly reduced wheat yield and quality. This reduction was reported due to the inactivation of the soluble starch synthase, a key heat-labile enzyme in starch transformation of wheat endosperm. To improve wheat productivity under heat stress, the rice soluble starch synthase I, under the control of either a constitutive promoter or an endosperm-specific promoter, was expressed in wheat and the transgenic lines were monitored for expression and the effects on yield-related traits. The results showed that the transgenic wheat events expressed rice soluble starch synthase I at a high level after four generations, and transgenic plants produced grains of greater weight during heat stress. Under heat stress conditions, the thousand kernel weight increased 21–34% in T_2_ and T_3_ transgenic plants compared to the non-transgenic control plants. In addition, the photosynthetic duration of transgenic wheat was longer than in non-transgenic controls. This study demonstrated that the engineering of a heat tolerant soluble starch synthase gene can be a potential strategy to improve wheat yield under heat stress conditions.

## Introduction

Wheat (*Triticum aestivum* L.) is one of the most important main food crops worldwide. It provides approximately 20% of the calories consumed by humans (Pfeifer *et al.* 2014). Given the socioeconomic importance of wheat, many efforts of breeding have focused on improving its yield, quality, and adaptability to biotic and abiotic stresses. Generally, wheat yield and quality can be affected by various environmental constraints such as high temperature, drought, or the combination of these two (Wardlaw *et al.* 2002; Stratonovitch and Semenov 2015). With global climate changes, the frequency of extreme high temperatures is expected to increase and negatively impact crop production, especially for cold season crops (Pradhan *et al.* 2012). It is reported that for every 1°C rise above the optimum temperature during the grain filling stage, wheat yield is reduced 3 to 4% (Wardlaw *et al.* 1989). Ever-increasing evidence in the past decades has demonstrated that elevated temperature during wheat anthesis and grain filling stages significantly reduces grain yield (Bhullar and Jenner 1986a; Keeling *et al.* 1993; Farooq *et al.* 2011; Prasad and Djanaguiraman 2014). Even short-term heat stress could cause an 11–23% reduction in grain number and 10–26% reduction in individual grain sizes (Talukder *et al.* 2013). In the Great Plains regions of the USA, high temperature stress is not uncommon during early reproductive development and grain filling periods of wheat when wheat is most susceptible to extreme temperatures (Assad and Paulsen 2002). Improving the heat tolerance in wheat has been considered a key goal that would result in yield stability (Kuchel *et al.* 2007).

A major component of wheat seeds is starch, which accounts for 55–75% of total dry grain weight (Gillies *et al.* 2012). Therefore, the grain yield depends largely on starch accumulation in the seed endosperm (Jenner *et al.* 1991). Several processes in starch deposition during the grain filling stage have been identified in response to heat stress. Starch granules in higher plants usually contain two classes of glucose polymers, amylose, and amylopectin. In wheat, amylose is a linear or less branched molecule with a low degree of polymerization comprising 25–30% of wheat grain starch, and amylopectin is a large and highly branched polymer comprising 70–75% of grain starch (Myers *et al.* 2000; James *et al.* 2003). Both amylose and amylopectin are synthesized from ADP-glucose (Myers *et al.* 2000). Soluble starch synthase (SSS) enzymes catalyze the extension of linear glucan chains by adding glucose units to the glucan non-reducing ends through α-1,4 linkages, and then starch branching enzymes cleave and reattach the glucan segments to form branches on the polymers (Jeon *et al.* 2010). In cereal endosperm, these genes have been divided into five classes, including the soluble SS I-IV (SSI, SSII, SSIII, and SSIV) and granule bound starch synthase (GBSS) (Hirose and Terao 2004).

Previous studies indicated that the adverse effects of elevated temperature on starch deposition in wheat grain endosperm are likely due to the inactivation of starch synthesis (Bhullar and Jenner 1986a, b; Rijven 1986; Jenner 1994; Zhao *et al.* 2008; Prakash *et al.* 2009) and that soluble starch synthase I was the most heat-labile enzyme limiting the starch deposition in the biosynthetic pathway in seeds (Keeling *et al.* 1993; Jenner 1994; Sumesh *et al.* 2008; Prakash *et al.* 2009). In the developing endosperm, the expression levels of starch biosynthesis genes have been also reported to be early induced, but significantly suppressed by heat stress (Hurkman *et al.* 2003). Genetic engineering of such key enzymes to increase their thermostability might be an alternative approach to improve the starch biosynthesis under heat stress conditions.

The soluble starch synthase genes from a subtropical species, rice (*Oryza sativa* L.), could be good candidates to be introduced into wheat by genetic engineering. Previous studies in rice have demonstrated that the predominant SSI was stable and active at high temperature up to 35°C, resulting in increased production of long chain of amylopectin in the endosperm (Jiang *et al.* 2003). The presence of either SSI or SSIIIa gene could sustain the starch biosynthesis in rice, and SSI may have higher activity than SSIIIa, accounting for almost 70% of the total SS activity (Fujita *et al.* 2006; Fujita *et al.* 2011). A similar phenomenon observed in maize, the purified SSI enzyme showed a higher heat stability than SSIII at an incubation temperature of 42°C (Huang *et al.* 2016). The rice SSI presents only one isoform in rice (Baba *et al.* 1993; Hirose and Terao 2004) and shares 81% amino acid similarity with wheat SSI that produces a 75-kDa protein (Li *et al.* 1999; McMaugh *et al.* 2014).

The present report evaluated the overexpression of the rice SSI gene in transgenic wheat. The transgenic plants had significantly enhanced grain yield under heat stress during the grain filling stage, likely due to higher starch deposition resulting in greater kernel weight compared to the non-transgenic wheat. These results demonstrate that engineering heat-sensitive key enzymes can be a useful strategy for improving the heat tolerance of cold season crops, and for developing food crops adapted to an increasingly changed environment.

## Materials and Methods

### Plant materials

A spring wheat, *T*. *aestivum* L. cv. ‘Bobwhite’, was used for transformation, as well as phenotypic comparison in this study. Plants were grown in a controlled environment with a 16-h photoperiod at 600 μmol m^−2^ s^−1^ (Sylvania 20934 T-5 fluorescent tube, Sylvania, Wilmington, MA). The day/night temperatures were set at 20/15°C. Immature embryos were separated from surface sterilized caryopses and placed the embryo axis faced down in contact with CM4 medium at 25°C for 1 wk in darkness for callus formation as described by Zhou *et al.* (1995). The caryopses were collected from wheat plants that were at the stage of 12–14 d post anthesis. After callus formation, the embryogenic calluses were selected and prepared for biolistic bombardment.

### Thermostability prediction

Relative thermostability of the rice SSI protein, the amino acid sequences of rice (UniPort database: Q0DEC8), and wheat SSI (Genbank Accession ID: AAD54661) was performed using a novel scoring algorithm as described by Li *et al.* (2010).

### Plasmid constructs

**Figure 1.**
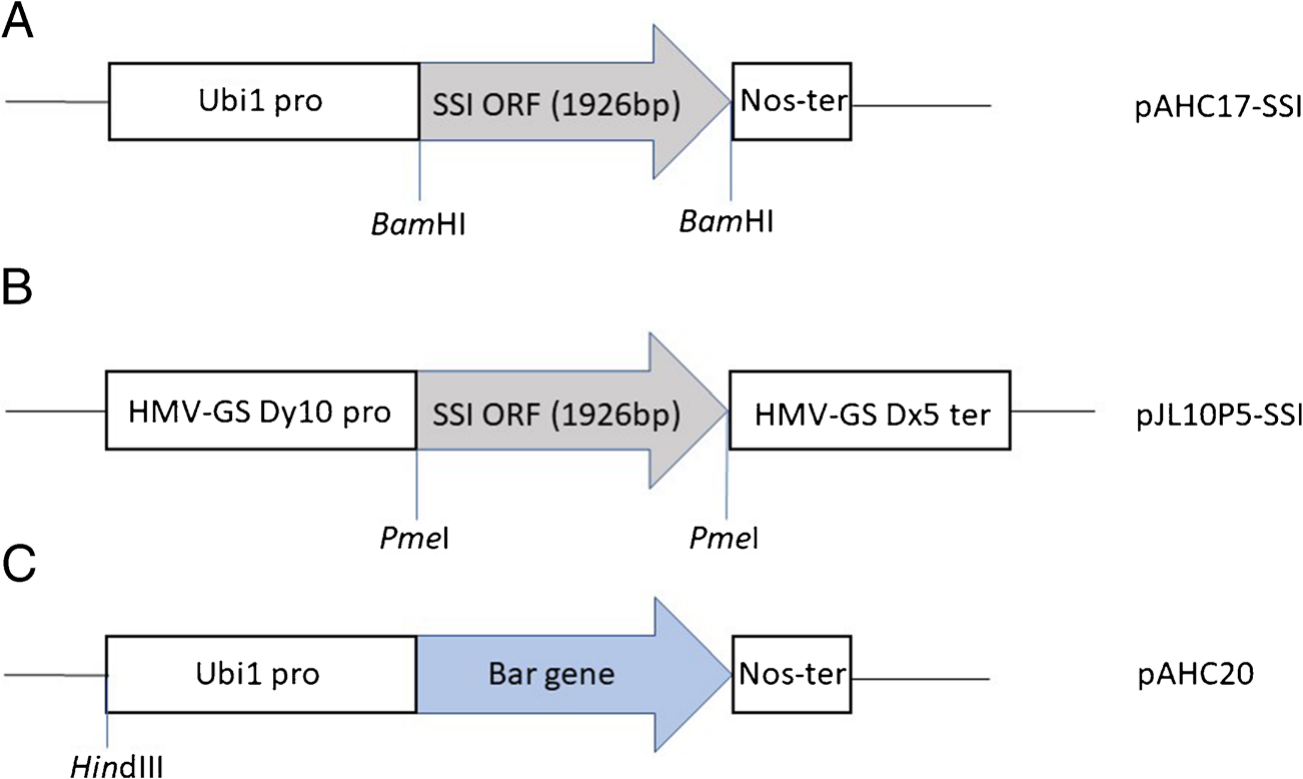
Schematic representation of the constructs for SSI and *bar* gene expression. (*A*) Construction map of pAHC17-SSI expression region. It contained the maize ubiquitin 1 promoter (Ubi1), the rice SSI gene, and nopaline synthase terminator (Nos-ter). The SSI ORF was sub-cloned into pAHC17 vector using BamHI restriction site. (*B*) Construction map of pJL10P5-SSI expression region. It contained Dy10 high-molecular-weight glutenin promoter (HMW-GS Dy10), the rice SSI gene, and the Dx5 terminator. The SSI ORF was sub-cloned into pJL10P5 using Pme I restriction site. (*C*) Construction map of pAHC20 containing *bar* gene which confers resistance to the herbicide Liberty® (glufosinate-ammonium).

The expression cassette in the pAHC17 vector contained the maize ubiquitin 1 (Ubi1) promoter (Christensen *et al.* 1992) and the nopaline synthase (NOS) terminator. The vector pJL10P5 (a kind gift from Dr. Ann Blechl, USDA-ARS, Albany, CA) was driven by endosperm-specific high-molecular-weight (HMW) glutenin Dy10 promoter (Lamacchia *et al.* 2001) and Dx5 terminator (Fig. 1*B*) from *T. aestivum*. The 1926-bp *O*. *sativa* soluble starch synthase I (SSI) ORF (Genbank accession ID: FJ750946) was subcloned into pAHC17 using a BamHI site and pJL10P5 using PmeI restriction sites (Fig. 1*A*, *B*). The orientation within the plasmid and the integrity of the SSI gene were confirmed by PCR and sequencing. The third construct, pAHC20 contained the bar gene controlled by Ubi1 promoter and NOS terminator (Fig. 1*C*) which confers resistance to glufosinate-ammonium, the active ingredient of the herbicide Liberty® (AgrEvo, Wilmington, DE).

### Wheat transformation

Biolistic transformation was performed using particle inflow gun as previously described by Finer *et al.* **(**1992). Plasmid DNAs (either pAHC17-SSI or pJL10P5-SSI) were mixed with pAHC20 in a 1:1 molar ratio prior to bombardment. After bombardment, all calluses were transferred to selective callus induction medium containing 5 mg L^**−**1^ glufosinate-ammonium and maintained in darkness for 2 wk. Recovery and regeneration of transgenic tissues was according to previously established protocols with slightly modification (Altpeter *et al.* 1996; Anand *et al.* 2003). The media used were described by Cheng *et al.* (1997). After 10–12 wk., T_0_ transgenic plants with developed roots and shoots were transferred into small peat pots for hardening with high humidity. These hardened plants were selected by herbicide resistance using 0.2% (*v/v*) Liberty® (AgrEvo) solution diluted with double-distilled water. The solution was applied using a cotton ball on adaxial leaves selected. The positive plants were transferred into Pro-Mix BX grow medium (Premier Tech, Rivière-du-Loup, Canada) and grown in the growth chamber 16 h day/8 h night, 500 μmol m^−2^ s^−1^ light intensity (Lucalux 400W High Pressure Sodium lamps, General Electric, Cleveland, OH), at 19°C.

### DNA extraction, polymerase chain reaction, and Southern analysis for transgenic wheat plants

In each generation, PCR testing was performed using genomic DNA to screen transgenic wheat plants for both *bar* gene and the rice SSI gene. Samples of 100–150-mg leaf tissue were taken and placed in a 2-mL centrifuge tube and homogenized with liquid nitrogen. Eight hundred-microliter 2× cetyltrimethylammonium bromide (CTAB) extraction buffer containing 4 μL 2-mercaptoethanol (Sigma-Aldrich®, St. Louis, MO) was added to each sample. Genomic DNA was isolated from leaf tissue using modified CTAB method described by Rogers and Bendich (1985). The presence of *bar* gene from pAHC20 was determined with a primer pair UbiAB-F (5′-CTTCAGCAGGTGGGTGTAGAGCGTG) and BarAB-R (5′-CCTGCCTTCATACGCTATTTATTTGC). The rice SSI-specific primer pair, osSSI-F (5′- CCCGATCTAGAAGGTCTCACAG), and osSSI-R (5′-TATGACAAGAACACTGCGGGC) amplified a 636-bp fragment, without any amplification of wheat genomic DNA and cDNA. The amplification condition was 95°C for 5 min, 32 cycles of 95°C 30 s, 60°C 30 s, and 72°C 30 s followed a final extension step at 72°C for 10 min. Southern blot analysis was conducted using a sequence amplified by osSSI-F and osSSI-R with pAHC17-SSI plasmid DNA as template. The PCR product was purified using QIAGEN® gel purification kit (QIAGEN®, Valencia, CA) and labeled with dCTP (α-^32^P) using Megapriming DNA labeling system (Amersham, Piscataway, NJ) following the manufacturer’s instructions. The labeled PCR product was used as a probe in Southern blot analysis. Twenty-five-microgram genomic DNA was digested with BamHI for Dy10 transgenic plants, and *Hin*dIII for both Ub1 and Ub2 transgenic lines. Digested DNA were separated on 0.8% agarose gels and transferred to a hybridization membrane. The membrane was probed with labeled PCR product (Sambrook and Russell 2006; Southern 2006).

### RNA extraction and reverse transcription polymerase chain reaction

Total RNA was isolated from both leaves and 20-d-old developing seeds of wheat. For the leaf sample, total RNA was isolated using RNeasy® Plant Mini Kit (QIAGEN®), while isolation from the seed was done by following a previously described protocol of Li and Trick (2005). Total RNA was treated with RQ1 DNase (Promega®, Madison, WI) to remove genomic DNA contaminants. One microgram of treated total RNA was used as template using a reverse transcription system kit (Catalog no. A3500, Promega®) following the product protocol. PCR was conducted using osSSI primers described above. DNA contamination was checked by performing PCR using the tubulin primers TubF:5′-ATCTGTGCCTTGACCGTATCAGG and TubR: 5′-GACATCAACATTCAGAGCACCATC. PCR conditions were the same as described above.

### Western blot analysis

Total soluble proteins in flag leaves and seeds were extracted from each T_2_ transgenic wheat event, one non-transgenic wheat variety (BW), and one rice variety (Nipponbare) at 20 d after anthesis. Proteins were extracted by grinding and homogenizing samples in protein extraction buffer (Fu *et al.* 2008). Protein concentrations were estimated by using Quick Start® Bradford Protein Assay kit (Bio Rad® Inc., Hercules, CA) according to manufacturer instructions. Samples were diluted in the buffer to maintain equal concentration for equal loading. Equal loading was also maintained by checking stained gels. Protein separation was performed by loading 40 μg of each sample on 10% (*w/v*) sodium dodecyl sulfate (SDS) polyacrylamide gels. SDS-PAGE separated proteins were then transferred to polyvinylidene difluoride (PDVF) membranes for immunoblotting. PDVF membranes with the transferred proteins were probed for rice SSI protein using rabbit polyclonal anti-SSI antibodies (AnaSpec, Inc., Fremont, CA), and goat anti-rabbit secondary antibodies conjugated to horseradish peroxidase (Santa Cruz Biotechnology, Inc., Santa Cruz, CA).

### Heat stress bioassays and phenotyping

A total of four independent heat stress experiments were conducted following similar procedure with different transgenic generations and temperature regimes. Day length of all the experiment was maintained as 16/8 h light/dark. The transgenic generations and high temperature regimes for all the experiments were as follows: Exp. 1 (T_2_ generation; 31/24°C day/night), Exp. 2 (T_2_; 33/26°C), Exp. 3 (T_3_; 34/28°C), and Exp. 4 (T_4_; 32–33/30°C). In each experiment, phenotyping was conducted under heat stress and optimum temperature conditions (22/15°C day/night for Exp. 1–3; and 20/15°C for Exp. 4), using eight to 20 pots for each event per line. Transgenic plants from ubiquitin promoter events (Ub-1, Ub-2), a Dy10 promoter event (Dy10) and non-transgenic Bobwhite produced from tissue culture (BWTC) were grown in the greenhouse. Following germination, seedlings were PCR screened for the presence of SSI gene in the laboratory and single seedlings were transplanted to pots (14 cm height, 50 cm top perimeter, and 36 cm bottom perimeter) filled with Metro Mix® 200 potting soil (Hummert™ Intl., Topeka, KS). The greenhouse was set at day/night temperature of 22/15°C with 16-h photoperiod supplemented with 400 W high-pressure sodium light (~ 800–600 μmol m^−2^ s^−1^) for normal growth. Plants were watered daily. One or two tillers per plant were tagged at anthesis, and half the plants from each event/line were transferred to a high-temperature growth chamber at 10 d after anthesis, based on the anthesis date of the tagged tillers. The remaining plants were transferred to another growth chamber having optimum day/night temperature (22/15°C) with the same light conditions as the high temperature chamber. Thousand kernel weight (TKW) was obtained from the tagged tillers by drying to equivalent moisture content, weighing the seeds and converting to TKW using the seed number of that tagged head. The number of effective tillers was also counted during harvest. Chlorophyll content was measured as described by Uauy *et al.* (2006), on flag leaves every other d for 12 d, starting at 12 d after anthesis. Days required for physiological maturity were calculated from date of anthesis to date of physiological maturity (based on 50% of stems turning yellow). Non-transgenic and transgenic event means were compared using two sample *t* tests with unequal sample variance at *α* = 0.05.

## Results and Discussion

### Comparison between wheat and rice SSI genes

**Figure 2.**
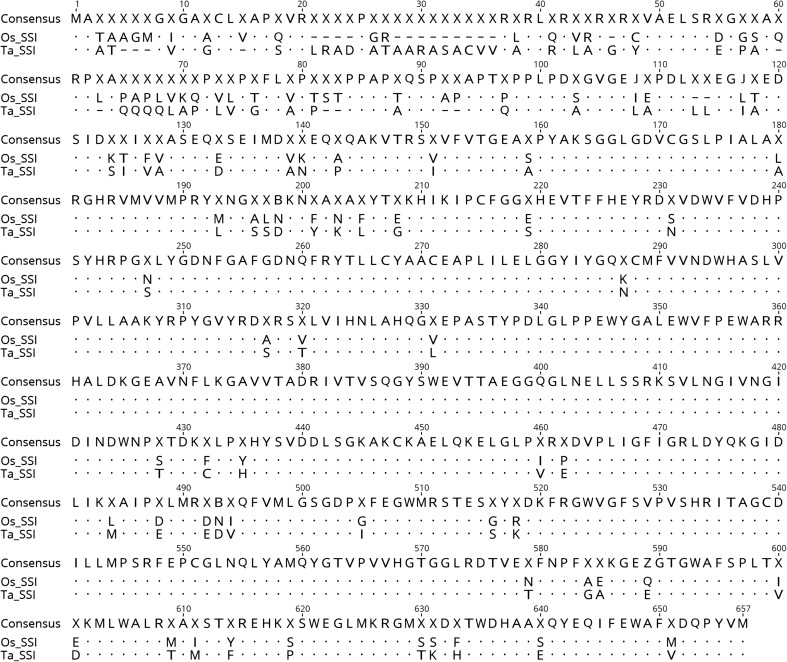
Amino acid sequence comparison for two SSI enzymes from wheat (Ta_SSI: 647aa) and rice (Os_SSI: 641aa). Two enzymes share 82% identity. *X* indicates amino acid mismatch, *dash* indicates gaps in alignment, and *bullet* indicates sequence alignment.

The SSI enzyme is responsible for the elongation of shorter A and B_1_ chains during starch biosynthesis in the soluble phase of the amylopectin (Commuri and Keeling 2001; Fujita *et al.* 2006) and therefore the inactivation or destruction of this gene can result in a significant impact on crystalline amylopectin matrix formation, as well as grain filling. To determine the relative thermostability of the rice SSI protein, the amino acid sequences of rice (UniPort database: Q0DEC8) and wheat SSI (Genbank Accession ID: AAD54661) were compared to each other (Fig. 2). Using a novel scoring algorithm to compare relative thermostability of proteins using an integrated statistical and machine learning approach (Li *et al.* 2010), it was predicted that the rice SSI protein was more thermostable than the wheat SSI protein (data not shown). This result agrees with previous reports that the wheat soluble SSI enzyme is heat-labile based on bioassays (Keeling *et al.* 1993; Jenner 1994; Sumesh *et al.* 2008; Prakash *et al.* 2009). To test the hypothesis that the expressed heat-stable rice SSI gene in wheat would improve the grain filling potential of wheat under moderate heat stress, the rice SSI gene was selected and introduced into the Bobwhite wheat variety.

### Overexpression of rice SSI gene in wheat and segregation patterns

Two constructed expression vectors, pAHC17-SSI and pJL10P5-SSI, were co-transformed into wheat with pAHC20 containing the selective marker gene *bar*. After transformation and herbicide selection, a total of six bar positive transgenic wheat events were identified. Five of six events contained the modified rice SSI gene as determined by PCR analysis from leaf DNA in the T_0_ generation (Table 1). Ultimately, three SSI-positive events were obtained for further analyses. Two transgenic events were under the control of Ubi1 promoter, assigned as Ub1 and Ub2. The third event was under the control of the wheat HMW-glutenin Dy10 promoter, assigned as Dy10, which was only expressed in seeds.

**Table 1.** Presence of rice SSI gene in six Liberty® positive *Triticum aestivum* L. cv. ‘Bobwhite’ events and their *Χ*^2^ values

Event no.	Promoter	PCR test in T_0_ plants	Number of PCR positive T_1_ plants	Number of PCR negative T_1_ plants	*Χ*^2^ value (based on 3:1 ratio)
1286 (Ub1)	Ubiquitin	+	37	6	2.79
1588	Ubiquitin	+	4	2	1.33
1700 (Ub2)	Ubiquitin	+	7	2	0.037
2036	DY10	+	46	20	0.99
2173 (Dy10)	DY10	+	13	5	0.074
2540	DY10	–	0	10	–

**Figure 3.**
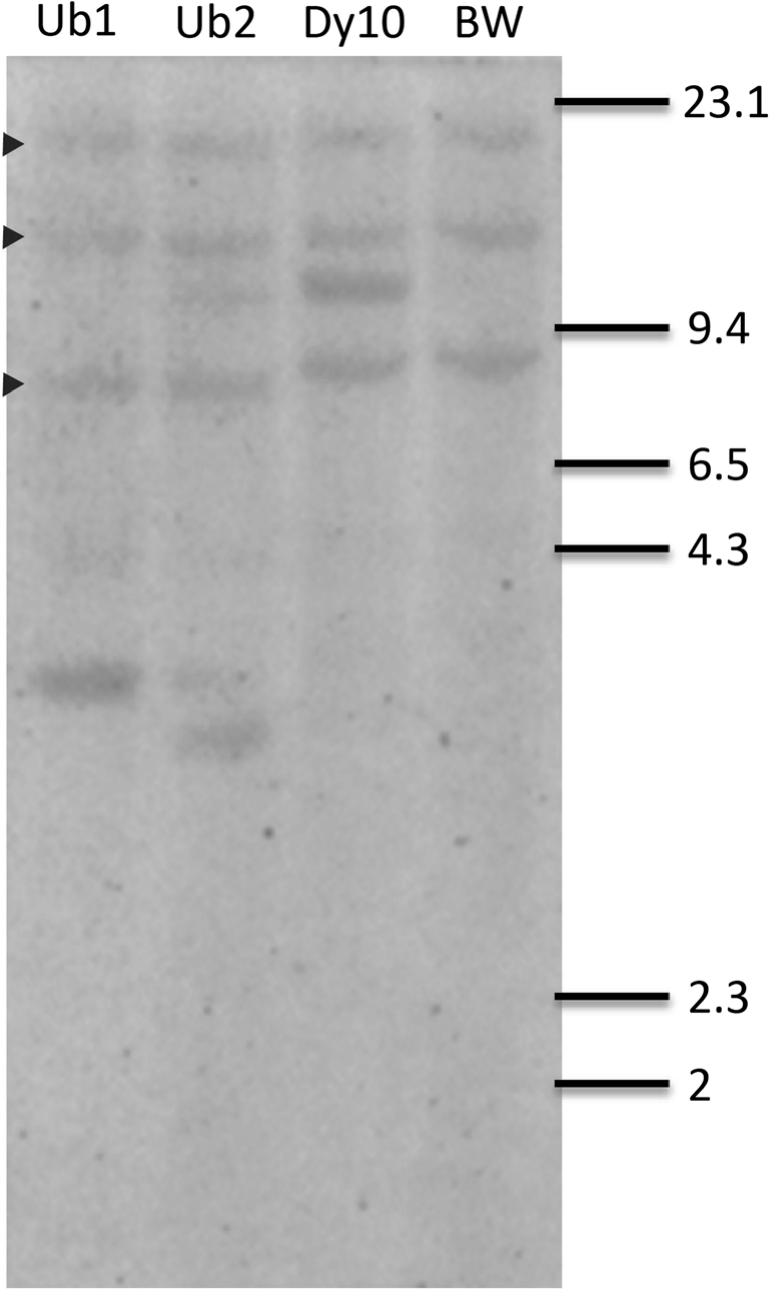
Southern blot analysis of three T_2_ generation transgenic *Triticum aestivum* L. cv. ‘Bobwhite’ events and a non-transgenic Bobwhite (BW) control. Twenty-five micrograms of leaf DNA was digested with BamHI for DY10, or HindIII for Ub1 and Ub2 events, fractioned on 0.8% agarose gel, transferred to a nylon membrane, and hybridized with 32p-labeled PCR product of rice SSI gene. Ub1, event number 1286 with ubiquitin promoter; Ub2, event number 1700 with ubiquitin promoter; Dy10, event number 2173 with Dy10 promoter; BW, non-transgenic Bobwhite variety. *Arrows* indicated the endogenous SSI genes in hexaploid wheat.

Analysis of SSI gene segregation using *Χ*^2^ test in T_1_ plants showed that there may be one or more transgene copies of the SSI in the events (Table 1). Southern analysis using genomic DNA from T_2_ leaves and the SSI gene sequence as a probe demonstrated that all three tested transgenic events and the non-transgenic BW had three bands in common (Fig. 3), indicating that the probe could be hybridized with three endogenous SSI genes, corresponding to each of the three genomes of hexaploid wheat. This observation is consistent with a previous report (Li *et al.* 1999) that high similarity (88%) between the rice and wheat SSI genes. Additional unique bands were presented in each transgenic line (Fig. 3), confirmed the transgene integration. Based on band numbers, Ub1 and Dy10 transgenic events appeared to harbor only a single SSI gene insertion (single copy), while Ub2 event was likely to harbor three copies of the rice SSI gene.

### Molecular analysis of transgenic wheat plants

**Figure 4.**
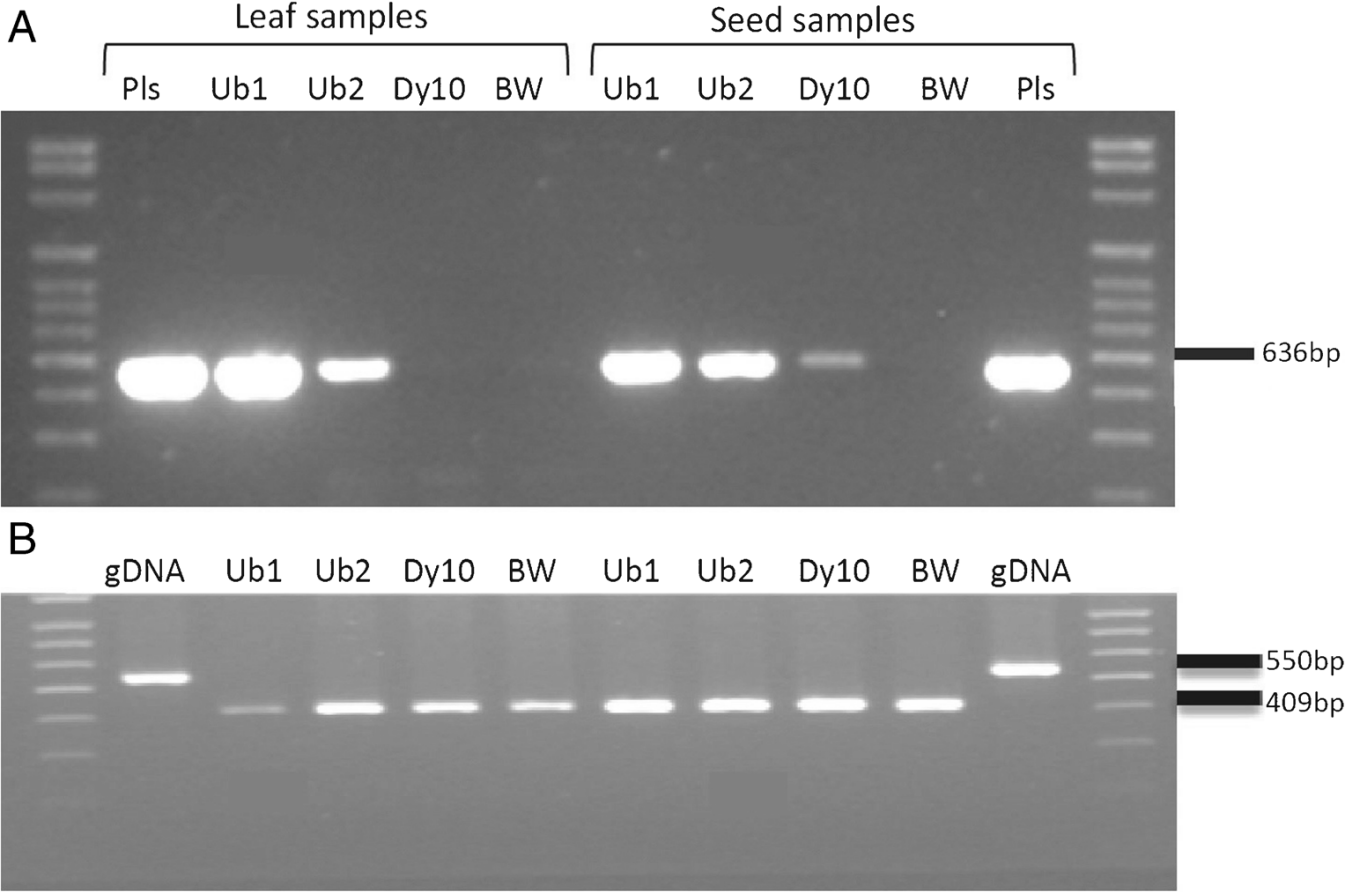
RT-PCR for leaf samples (*left*) and seed sample (*right*) of *Triticum aestivum* L. cv. ‘Bobwhite’. (*A*) Total RNA was isolated from both leaf and seed samples, and was used to carry out RT-reaction, following PCR was done with SSI-specific primers and visualized on 1% agarose gel. Pls plasmid DNA having SSI gene, Ub1 event number 1286 with Ubi1 promoter, Ub2 event number 1700 with Ubi1 promoter, Dy10 event number 2173 with Dy10 promoter, BW non-transgenic *Triticum aestivum* L. cv. ‘Bobwhite’. (*B*) RT-PCR with corresponding cDNA samples using tubulin gene primers for leaf samples (*left*) and seed sample (*right*). The tubulin primers amplified a 409-bp band for cDNA, and 550-bp band for genomic DNA, separately. gDNA genomic DNA of BW.

To confirm the transgene expression in wheat plants, RT-PCR was conducted using total RNA isolated from both leaf and seed samples of the three transgenic lines and non-transgenic Bobwhite. The leaf samples from Ub1 and Ub2 produced a band matching the expected size from the rice SSI gene, while the Dy10 event and the non-transgenic BW did not show any bands (Fig. 4*A*). The failure of the Dy10 event to produce a band using cDNA from the leaf sample was due to the tissue specificity of the Dy10 promoter as expected. The seeds of non-transgenic BW did not show any bands, while the seed samples of all three independent events showed expected rice SSI bands (Fig. 4*A*). It confirmed the expression of the rice SSI gene driven by both Ubi1 and Dy10 promoters in the transgenic wheat. Single tubulin bands with different sizes between genomic DNA samples and RNA samples demonstrated that RNA samples were not contaminated with DNA (Fig. 4*B*).

**Figure 5.**
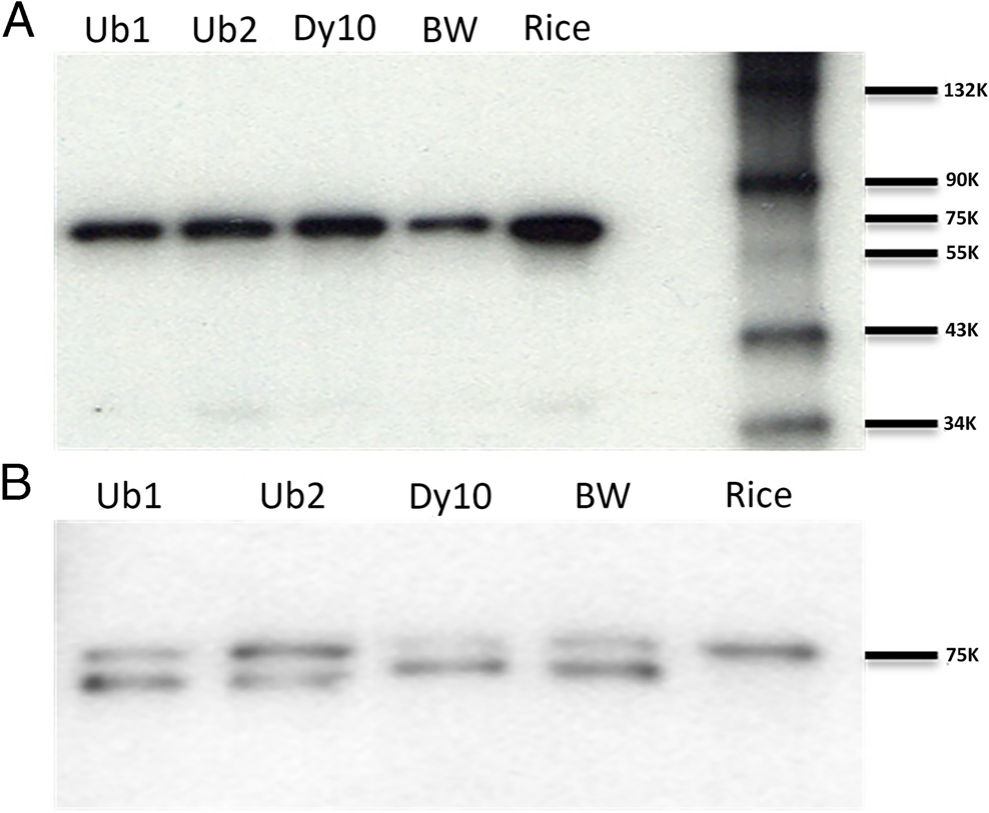
Western blot analysis of seed and leaf samples from T_2_ generation of *Triticum aestivum* L. cv. ‘Bobwhite’. Three T_2_ generation transgenic events, one non-transgenic *T. aestivum* L. cv. ‘Bobwhite’ and one rice sample was used. Total proteins extracted from both leaf and seed samples were fractionated on 10% sodium dodecyl sulfate-polyacrylamide gel (SDS page gel) and transferred to PDVF membrane, probed with polyclonal antibody raised against rice SSI. Equal amount of total protein (40 μg) was loaded in each lane. In seed samples (*A*), all transgenic events along with rice showed stronger signal than non-transgenic wheat. The antibody bound with both wheat endogenous SSI and rice SSI protein. In leaf sample (*B*), antibody bound with two SSI isoforms of wheat. Comparing the signal strength of the second isoforms of SSI, Ub1, Ub2, and rice leaf sample showed enhanced signal for SSI.

Western blot analysis was conducted to detect the presence of SSI proteins in wheat. A rice leaf sample was included as a control. In western blot analysis, the differentiation between rice SSI from wheat SSI was not successful as the antibody bound to both rice and wheat proteins (Fig. 5). This cross-reactivity was likely due to the high similarity of the amino acid sequences and length between the two proteins (Fig. 2). In seeds, only one SSI protein band was detected in the three transgenic events, non-transgenic BW, and rice. The SSI proteins showed a similar molecular weight (71 kDa) in both wheat and rice with stronger intensities in the transgenic wheat events than in non-transgenic wheat (Fig. 5*A*), indicating that the rice SSI protein was probably produced in the endosperm of all three transgenic wheat events. A previous study showed that the rice SSI protein produced a 57-kDa protein band (Baba *et al.* 1993). However, in the present study, an approximate 71-kDa band was detect by the western blot, which was similar to the 71-kDa wheat SSI band. The cDNA sequence of the rice SSI was used to query the UniProt database (www. Uniport.org), and the expected size of the protein (accession no. Q0DEC8) of 70.95 kDa was predicted, which was supportive of this current finding.

In leaves, two SSI protein bands (isoforms 60 and 71 kDa) were detected in three transgenic events and non-transgenic BW. The 71-kDa SSI isoform showed stronger band intensity in the Ub2 event than in the Ub1 event, Dy10, and non-transgenic BW. However, the 50-kDa SSI isoform showed weaker band intensity in the Ub2 even than in the Ub1 event, Dy10, and non-transgenic BW (Fig. 5*B*). The results indicated that there were probably two isoforms of SSI in wheat leaf tissue, but only one isoform existed in seeds. This observation is consistent with a previous report that two SSI isoforms were expressed in wheat leaf tissues, and only one isoform in seeds (Denyer *et al.* 1995). Only the 71-kDa isoform was detected in rice leaf tissue (Fig. 5*B*).

### The effects of elevated temperature on transgenic wheat plant

Moderately high temperatures reduce the SSI activity of wheat at both the transcription (Hurkman *et al.* 2003) and enzyme activity levels (Rijven 1986), while the reduction of transcription and enzyme activity in rice is mild (Yamakawa *et al.* 2007). Accordingly, the transgenic wheat plants in the present study were placed under the elevated temperature to determine if the thermostable rice SSI could compensate for the low activity of wheat SSI during heat stress. Successful compensation would be expected to increase the individual grain weight by sustaining the enzyme activity. Based on previous molecular analysis above, all three events (Ub1, Ub2, and Dy10) positive for transgene (SSI) were used for heat stress bioassays. The PCR negative plants from different transgenic events (BWTC) and Bobwhite plants not derived from tissue culture (BWNTC) were used as controls.

**Figure 6.**
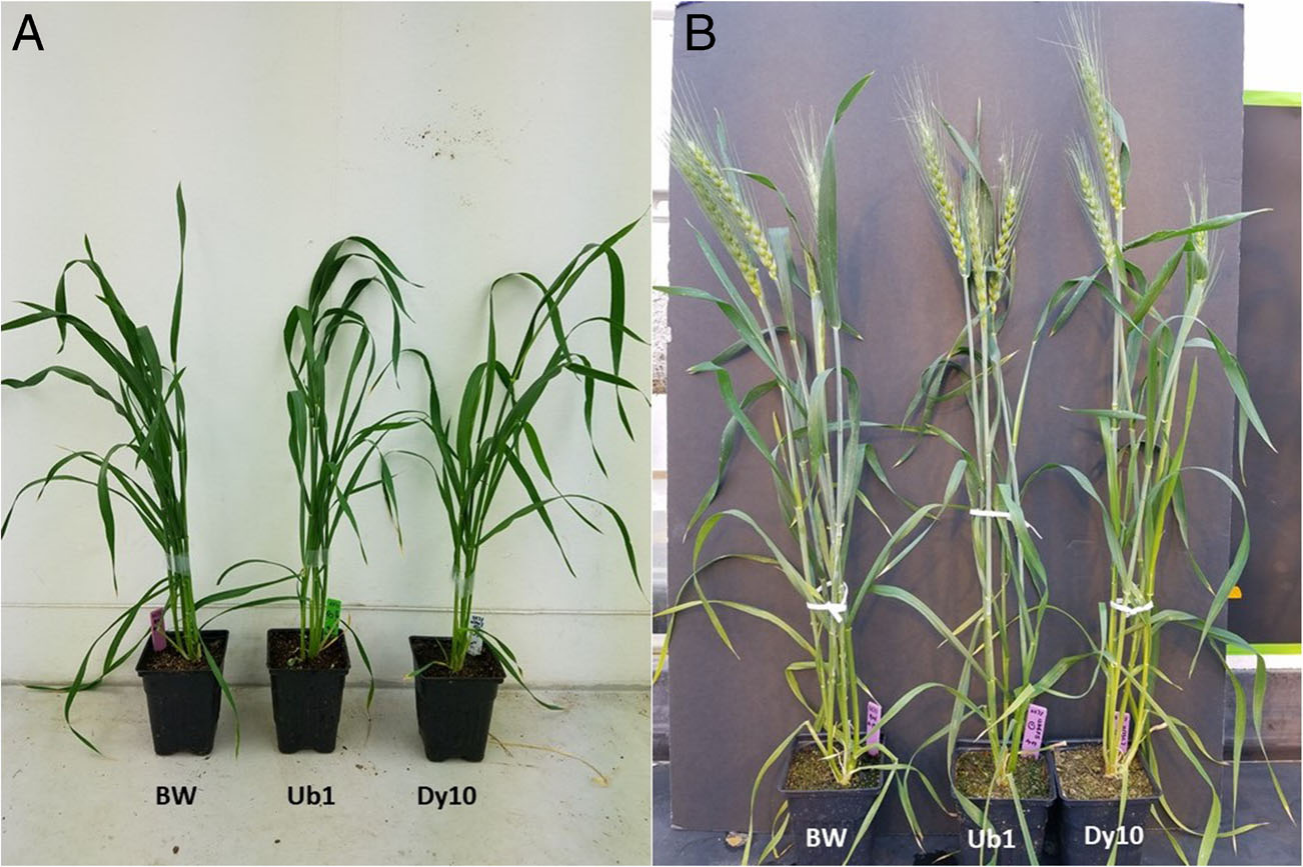
Phenotype comparison of transgenic lines and non-transgenic control lines. Plants were growing under optimum temperature. The transgenic lines showed similar phenotypes as non-transgenic control. Photos were taken at 40 d after germination (*A*), and 50 d after germination during *Triticum aestivum* L. cv. ‘Bobwhite’ heading stage (*B*).

Three independent experiments were conducted for phenotyping under increasing temperature ranges (31/24, 33/26, and 34/28°C with 16/8 h day/night) compared to optimal temperature conditions (22/15°C). In Exp. 1, T_2_ plants from Ub1, Dy10, and BWTC were exposed to heat stress at 31/24°C (day/night) from 10 d of anthesis until harvest. Significant differences were found for TKW for Ub1 (3.22%) and Dy10 (6.71%) events compared to BWTC (Table 2). Effective tiller number per plant and days required for physiological maturity showed no significant difference, but seed number per selected head was significantly lower for the Dy10 event (Table 2). Under optimum temperature conditions, there was no significant difference between transgenic and non-transgenic events for any studied traits (Fig. 6).

**Table 2.** Comparison of agronomic traits between transgenic plants and non-transgenic *Triticum aestivum* L. cv. ‘Bobwhite’ in heat stress Exp. 1

Temp.	Event name†	TKW (g)		Seed number per selected head	
		Mean ± STD	T stat	Mean ± STD	T stat
31/24°C	BWTC	29.5 ± 1.21		54.33 ± 11.58	
	Ub1	30.45 ± 1.32	1.85*	54.2 ± 14.24	0.02
	Dy10	31.48 ± 0.83	3.47**	41.25 ± 4.03	2.54*
22/15°C	BWTC	35.62 ± 1.5		59.33 ± 11.5	
	Ub1	37.71 ± 1.96	1.92	58.55 ± 11.1	0.1
	Dy10	37.53 ± 2.89	1.13	45.75 ± 12.44	1.49

*TKW* thousand kernel weight, *BWTC* non-transgenic Bobwhite but established through tissue culture, Ub1 event 1286 having ubiquitin promoter, Dy10 event 2173 having Dy10 promoter, *STD* standard deviation

†Number of plants studied in this experiment is accompanied by event name in parenthesis

*, **, and *** denote *P* < 0.05, 0.01, and 0.001 respectively

**Figure 7.**
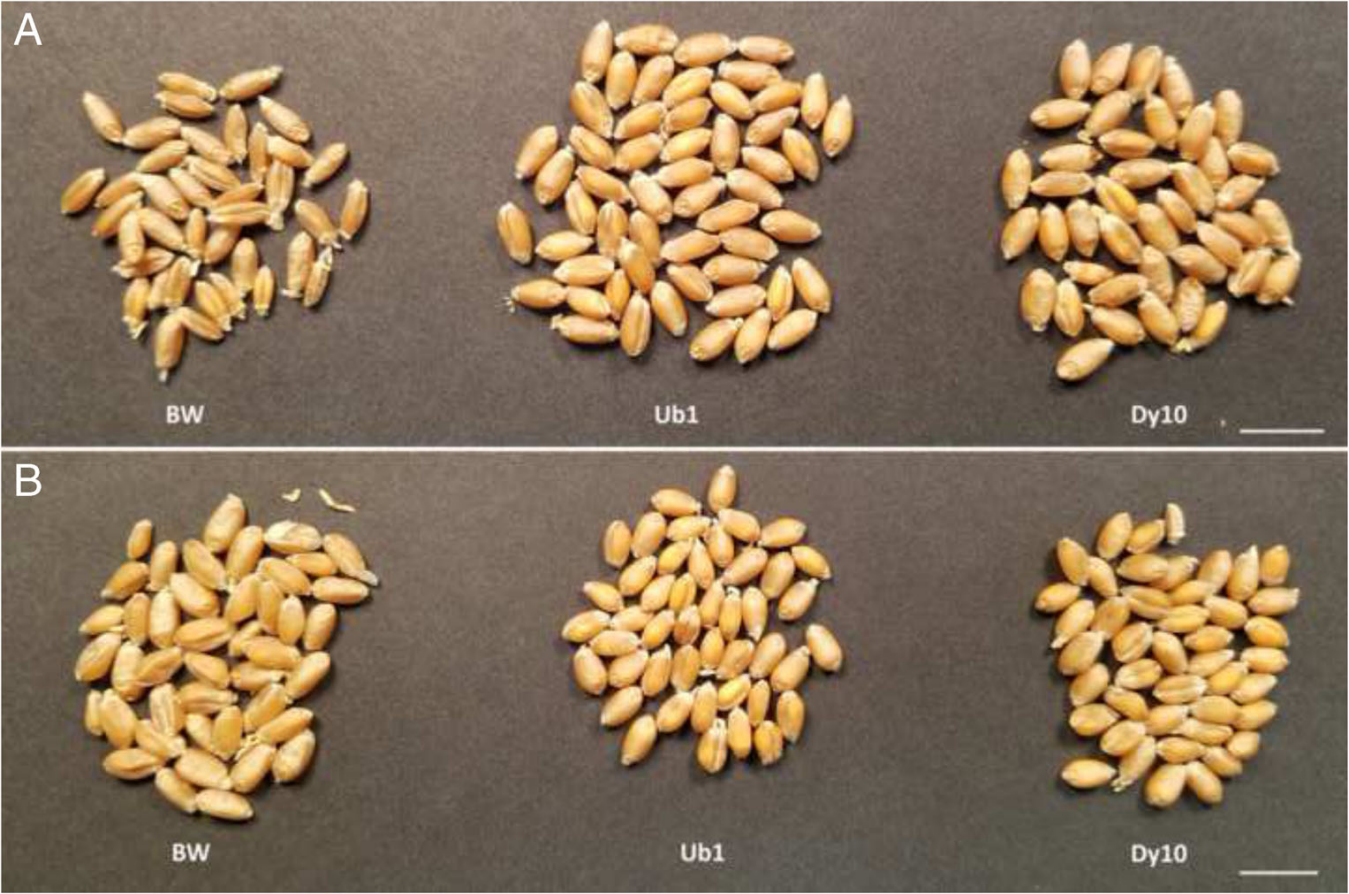
*Triticum aestivum* L. cv. ‘Bobwhite’ seed phenotype comparison under heat stress and normal temperature. (*A*) Harvested seeds were produced by plants grown under elevated temperature (34/28°C) after anthesis. The seeds of non-transgenic plants (BW) were visually smaller than seeds of transgenic lines (Ub1 and Dy10). (*B*) Harvested seeds were produced by plants grew under optimal temperature condition (22/15°C). There was no significant difference among seed size of transgenic seeds (Ub1 and Dy10) and non-transgenic controls (BW). The scale bars represent 1 cm.

In Exp. 2, an increased temperature (33/26°C) was used for all three (Ub1, Ub2, and Dy10) transgenic events. Both BWTC and BWNTC were pooled as controls since there were no significant differences observed for the traits evaluated under heat and normal growing conditions. Transgenic events with the Ub1 (10.70%) and Dy10 (25.67%) promoters showed significant increases in TKW compared to control plants (Table 3). Individually, all events with the transgene had higher TKW than non-transgenic lines. The Dy10 event produced significantly lower number of seeds than non-transgenic control (BW) in both heat stress (33/26°C) and optimum temperature (22/15°C) conditions (Table 3). This result could be indicative of an interaction between the Dy10 promoter and seed setting capability, or simply the effect of somaclonal variation, which might have been fixed with advancing the generation. There was no evidence in the previous literatures suggesting that the Dy10 promoter would compromise seed setting capability. Additional events with the Dy10 promoter may be necessary to draw a more definite conclusion. Under optimum temperature conditions, tiller number and days required for physiological maturity were not significantly different (Table 3). Moreover, the Dy10 event took significantly longer to reach physiological maturity (32.66 d) under heat stress than non-transgenic events. It has been reported that pulling sugar from the leaf to seed helps to reduce feedback inhibition of leaf sugar on photosynthesis (Smidansky *et al.* 2002). It is possible that altered source-sink relationships resulted in a longer grain fill period, although additional events would need to be examined to draw a firm conclusion. In Exp. 3, an even higher temperature regime (34/28°C) was used in the bioassays with T_3_ plants. Under heat stress condition, transgenic events with both Ubi1 (22.94%) and Dy10 (34.59%) promoters produced seeds having significantly higher TKW (Tables 3 and 4) and visibly larger kernels than non-transgenic events under heat stress condition (Fig. 7). Comparably, the Dy10 event in T_3_ generation still produced lower number of seeds per selected head, while Ub1 and Ub2 events showed no difference compared to controls (Table 3). Tiller number per plant and chlorophyll content was not significantly different between transgenic and non-transgenic events (data not shown). Days required for physiological maturity differed significantly between transgenic and non-transgenic events, with transgenic events averaging 1.28 additional days of grain filling duration than non-transgenic plants. This observation infers the photosynthetic duration of the heat-stressed plants were longer than non-transgenic control plants.

**Table 3.** Comparison of agronomic traits between transgenic and non-transgenic *Triticum aestivum* L. cv. ‘Bobwhite’ in Exp. 2 and Exp. 3 at different temperature ranges

Temp.	Event name	TKW				Seed numbers per selected head				Physiological maturity (days)			
		2nd experiment (high temp 33/26°C)		3rd experiment (high temp 34/28°C)		2nd experiment (high temp 33/26°C)		3rd experiment (high temp 34/28°C)		2nd experiment (high temp 33/26°C)		3rd experiment (high temp 34/28°C)	
		Mean ± STD	T stat	Mean ± STD	T stat	Mean ± STD	T stat	Mean ± STD	T stat	Mean ± STD	T stat	Mean ± STD	T stat
High temperature	BWTC	22.87 ± 1.94	0.301	20.23 ± 2.65	0.134	47.40 ± 4.56	0.48	46.6 ± 11.74	0.32	26.8 ± 3.27	0.25	22.8 ± 1.30	0.63
	BWNTC	22.51 ± 1.67		19.94 ± 3.89		46.00 ± 4.24		48.8 ± 9.73		27.25 ± 2.22		22.4 ± 0.54	
	BW	22.71 ± 1.73	–	20.09 ± 3.14	–	46.78 ± 4.20	–	47.7 ± 10.23	–	27 ± 2.69	–	22.6 ± 0.97	–
	Ub1	24.45 ± 1.44	1.89*	24.35 ± 4.31	1.96*	46.00 ± 4.08	0.31	50.4 ± 12.80	0.41	29 ± 3.92	0.93	23.4 ± 0.89	1.59
	Ub2	25.17 ± 2.35	1.88*	25.03 ± 2.46	3.50**	49.50 ± 13.47	0.40	48.33 ± 4.13	0.17	28 ± 2.45	0.66	23.66 ± 0.82	2.36*
	Ub	24.81 ± 1.84	2.41*	24.70 ± 3.24	3.30**	47.75 ± 9.41	0.27	49.27 ± 8.67	0.38	28.5 ± 3.07	1.06	23.54 ± 0.82	2.41*
	Dy10	28.54 ± 1.85	6.14***	27.04 ± 3.12	3.75**	36.83 ± 9.22	2.48*	25.25 ± 5.85	5.14**	29.66 ± 2.50	1.96*	24.25 ± 0.96	2.90*
Optimum temperature 22/15°C	BWTC	40.95 ± 2.27	0.144	40.91 ± 2.68	0.932	39.0 ± 8.18	0	50.2 ± 11.26	0.989	40.8 ± 1.79	1.31	38.8 ± 1.30	0
	BWNTC	40.69 ± 3.00		39.45 ± 2.25		39.0 ± 10.61		56.4 ± 8.35		43 ± 2.94		38.8 ± 4.82	
	BW	40.84 ± 2.41	–	40.18 ± 2.46	–	39.0 ± 8.70	–	53.3 ± 9.9	–	41.78 ± 2.49	–	38.8 ± 3.33	–
	Ub1	42.11 ± 3.37	0.68	43.57 ± 4.28	1.77	37.5 ± 9.15	0.276	53.83 ± 10.06	0.103	42 ± 3.46	0.115	36.5 ± 4.42	1.10
	Ub2	40.27 ± 1.36	1.81	43.17 ± 2.16	2.55*	49.5 ± 11.15	1.67	53.84 ± 9.54	0.106	41 ± 3.37	0.414	37.17 ± 5.19	0.69
	Ub	41.20 ± 2.57	0.292	43.38 ± 3.25	2.63**	43.5 ± 20.06	0.587	53.83 ± 9.35	0.129	41.5 ± 3.21	0.20	36.83 ± 4.61	1.16
	Dy10	40.53 ± 1.76	0.276	44.68 ± 4.54	1.87	30.0 ± 7.11	1.96*	36.75 ± 16.52	1.87	41.2 ± 1.64	0.52	40.25 ± 4.03	0.64

The Ub observation combines the Ub1 and Ub2 observations

*TKW* thousand kernel weight, *BWTC* non-transgenic Bobwhite established through tissue culture, *BWNTC* non-transgenic Bobwhite not regenerated through tissue culture, Ub1 event 1286 having ubiquitin promoter, Ub2 event 1700 having ubiquitin promoter, Dy10 event 2173 having Dy10 promoter, *STD* standard deviation

*, **, and *** denote *P* < 0.05, 0.01, and 0.001 respectively

**Table 4.** Comparison of *Triticum aestivum* L. cv. ‘Bobwhite’ thousand kernel weight (TKW), TKW percent change, and mean seed number at high temperature and optimum temperature conditions

Experiment	Event	TKW		% TKW increment	Seed number per selected head	
		Mean ± STD	T stat		Mean ± STD	T stat
Heat stress (32–33/30°C)	BW	23.55 ± 4.88			47.17 ± 8.7	
	Ub1	26.75 ± 2.55	1.39*	13.58	52.6 ± 1.95	1.48
	Ub2	26.39 ± 3.89	1.11	12.06	51.17 ± 6.55	0.90
	Dy10	30.50 ± 3.38	2.94**	29.51	46.57 ± 3.78	0.16
Optimum temperature (20/15°C)	BW	34.27 ± 2.10			47.0 ± 12.0	
	Ub1	34.72 ± 1.00	0.43	1.31	50.4 ± 10.41	0.47
	Ub2	35.21 ± 0.78	0.95	2.74	49.83 ± 9.26	0.43
	Dy10	37.59 ± 1.21	2.97	9.67	49 ± 10.1	0.27

*STD* standard deviation

* and ** denote *P* < 0.05 and 0.01 respectively

In Exp. 4, the heat stress bioassay with a higher nighttime temperature and lower daytime temperature (32/33°C) was also performed. Similar to previous results, Ub1 (13.58%), Ub2 (12.06%), and Dy10 (29.51%) showed considerable increase in TKW than non-transgenic controls under high temperature (Table 4). There was high variability in kernel weight within the events; as a result, even though there was a remarkable increment in Ub1 and Ub2, they were not significantly different. None of the transgenic events showed significant variation for seed number per head in any temperature condition, indicating seed set was not affected. This result indicates such differences are probably due to starch deposition rather than compensation for reduced seed set.

**Figure 8.**
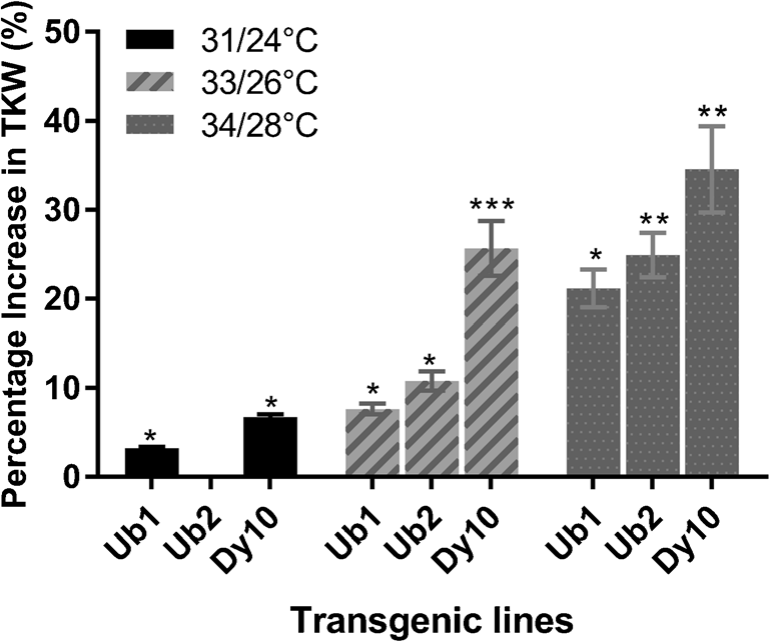
Percent change in thousand kernel weight (TKW) compared to control for three independent experiments using *Triticum aestivum* L. cv. ‘Bobwhite’. The Ub2 was absent for the 31/24°C treatment. *, **, and *** denote *P* < 0.05, 0.01 and 0.001 respectively found in the comparison.

Taken together, three transgenic lines produced significantly heavier seeds compared to non-transgenic lines under heat stress in three independent experiments. This data suggests that starch synthase activity was maintained under high temperature and likely allowed continuous starch accumulation. The increased grain fill duration observed at optimum temperature may be due to reduction of feedback inhibition of photosynthesis by reducing the accumulation of sugars. Under optimum temperature, some transgenic events produced slightly heavier seeds (but not significantly) compared to controls. This observation may be due to the additive effects with the rice SSI transgene, or, more likely, may indicate other wheat starch synthase genes are affected by temperature fluctuations within the growth chambers. Nonetheless, these experiments demonstrate SSI is likely the most important enzyme for sustaining starch synthesis under heat stress. There was no significant variation among transgenic and non-transgenic events in optimum temperature conditions. It was clear that the TKW differences between the transgenic and non-transgenic lines increased (Fig. 8) as the temperatures were increased. These results support the hypothesis that wheat SSI enzyme is less stable than rice SSI at high temperature, and the additional stable rice SSI expressed in wheat is able to reduce the affect in grain yield by heat stress. It also supports previous research regarding the importance of SSI activity related to grain filling (Prakash *et al.* 2009). No significant variation in chlorophyll content was observed between transgenic and non-transgenic events, indicating that differences in starch deposition were not due to the differences in photosynthetic capacity. This result is in line with a previous report that reduced grain filling at high temperature was not likely due to the supply of the assimilate from photosynthesis (Sofield *et al.* 1977).

## Conclusion

With global warming, abiotic stresses, especially heat and drought, could be a critical limiting factor for crop production. These results indicate that introduction of thermostable SSS in wheat could be a useful strategy for improving heat tolerance. This approach might be beneficial for other cool season species that are grown under conditions where heat stress may limit production. In addition, it is useful to explore the heat stability of SSS from other species typically grown under high-temperature conditions to identify the most heat-stable sources of the enzyme. It is also very useful to explore genetic variability within the wheat gene pool for the ability to maintain greenness under heat stress (Ristic *et al.* 2008). Research on heat tolerance in wheat has also focused on the ability to retain green leaf area under stress. This ability to retain photosynthetic capacity under stress is important. It may be necessary to identify and utilize heat-stable starch synthase enzymes to be able to fully take advantage of genetic mechanism that retain green leaf area and preserve photosynthetic function. The observation of increased grain fill of transgenic plants under the highest stress levels further indicates the connection between these traits needs to be explored more fully. New biotechnology, coupled with knowledge on starch metabolism, will help provide new strategies for improving the heat stress tolerance in wheat grains.
